# Gender differences underlying the link between exposome and psychosis

**DOI:** 10.1192/j.eurpsy.2023.114

**Published:** 2023-07-19

**Authors:** S. Guloksuz

**Affiliations:** ^1^Psychiatry, Maastricht University, Maastricht, Netherlands; ^2^Psychiatry, Yale School of Medicine, New Haven, CT, United States

## Abstract

**Abstract:**

To investigate gender-related differences in the connection between psychosis and exposome, we conducted a systematic review and retrieved 47 research publications in the PubMed database that examined the association of psychosis with childhood adversity, substance use, urbanicity, migration, season of birth, and obstetric complication. The results show that childhood abuse may be more significantly related with psychosis risk and an earlier age of onset in women than in men. In addition, childhood adversity has been linked to the severity of different symptom dimensions in men and women. Urban upbringing and immigration are much more strongly related to psychosis risk in men than in women. Despite the higher prevalence of substance abuse comorbidity in men with psychotic disorders, it seems that the relationship between substance abuse and psychosis risk is stronger in women. Due to several methodological limitations, the small number of studies, and the lack of consistency across studies, these findings should be regarded with care. Overall, although further research is required, it appears that there are gender-related differences in the relationships between environmental exposures and psychosis. There is an urgent need to gain insight into the gender-related patterns underlying the association between psychosis and exposome. Future studies should thus go beyond considering gender only as a covariate and study gender as a possible effect-moderating factor.

**Disclosure of Interest:**

None Declared

